# Lack of adequate sun protection for children with oculocutaneous albinism in South Africa

**DOI:** 10.1186/1471-2458-8-225

**Published:** 2008-06-30

**Authors:** Patricia M Lund, Julie S Taylor

**Affiliations:** 1 Department of Biomolecular and Sport Science, Faculty of Health and Life Sciences, Priory Street, Coventry, UK; 2 School of Nursing and Midwifery, University of Dundee, 11 Airlie Place, Dundee, UK

## Abstract

**Background:**

Childhood is a high risk time for ultraviolet induced skin damage as this age group has more time and opportunity to be outdoors in the sun. Children in Africa with the inherited condition oculocutaneous albinism (OCA) are especially vulnerable due to their lack of protective melanin. They are highly susceptible to developing skin lesions that have both cosmetic and health complications, with a high risk of developing skin cancers. The study aimed to explore the adequacy of sun protection strategies of children with albinism in order to inform future provision.

**Methods:**

Community based participatory research methods were employed to investigate sun protection strategies in 90 pupils with OCA (40 female and 50 male) boarding at a special school educating pupils with visual impairment in a rural area of northern South Africa. Hats worn and sunscreen preparations used were examined during semi-structured face to face interviews conducted in small peer groups. The resident nurse interpreted if necessary and provided additional information on monitoring and treatment of skin lesions.

**Results:**

Participants with albinism in this study were exposed to high levels of ultraviolet radiation throughout the year and showed skin damage despite wearing protective head gear. All except one pupil possessed at least one hat, with a mean brim width of 5.4 cm. Gender differences in sun avoidance behaviour were documented, with females seeking shade during recreational periods and males playing soccer outside. Although 38% of pupils were using a sunscreen with a sun protection factor (SPF) rating, only 12% had government sponsored tubes of SPF15 cream. Government sponsored sunscreen preparations were only provided if actively sought, involving time consuming trips to regional hospitals, with inadequate availability and insufficient supply.

**Conclusion:**

Children with albinism living away from home in rural areas appear to have inadequate sun protection strategies. Changes in health policy could address these deficiencies. We recommend providing more detailed health care information, giving advice on appropriate styles of hat to wear and how to assess commercial SPF products. Health promotional material should also be evaluated to determine its effectiveness among user groups.

## Background

Oculocutaneous albinism (OCA) is a congenital condition causing hypopigmentation of the hair, skin and eyes. Diagnosis in a black population is clear and unequivocal. The distinctive phenotype of those affected with their pale, chalky, de-pigmented skin, sandy coloured hair and blue to hazel eyes marks them as 'different' in this population group, leading to problems of social integration and ostracism [1]. OCA2, the form of albinism found in Africa, is inherited as an autosomal recessive disorder. In this type of OCA tyrosinase, the key enzyme for synthesising melanin, is present and functional, as the gene encoding this enzyme is not mutated. The defect in OCA2 is in the P gene coding for a membrane protein of the melanosome, leading to malfunction of the melanin synthesis pathway [2].

Albinism is the major cause of childhood visual impairment in northern South Africa where the frequency is 1 in 1500 [3] to 1 in 1900 among the black population [4]. In addition to poor vision, UVR induced skin damage is a major health issue for this group. They are highly susceptible to sunburn, photo-ageing of the skin, solar keratoses and carcinomas [5,6]. Squamous cell carcinomas of the head and neck are the most common cutaneous tumours seen in African patients with albinism [7]. Longer term chronic consequences included wrinkling of the skin and solar elastosis [8]. In Tanzania chronic skin damage was found in all those with albinism by 12 months of age [9].

Solar radiation is a human carcinogen. Actinic keratoses, pink, scaly spots, are pre-cursors to skin cancers and an indicator of cumulative UVB exposure. These are commonly found in middle aged to elderly fair skinned people living in sunny areas such as Australia and South Africa. In the black population with albinism in Africa, they occur in young children. Nonmelanoma skin cancers including basal and squamous cell carcinomas, while rare in black populations [10], are common among the susceptible group with albinism [11]. These cancers increase morbidity, particularly in equatorial regions of Africa, are disfiguring and expensive to treat. Studies in west, east and southern Africa reveal the extent of the vulnerability of those affected by albinism. In Nigeria, OCA patients as young as 20 years old developed basal cell carcinomas [12] leading to a shortened lifespan [13]. Dermatologists in Tanzania examining 164 patients with albinism found skin cancers in 25% of those aged over 20 [14]. In a South African study of 111 individuals with OCA the frequency of skin cancer was 23%, with the head the most commonly affected site [6]. The extreme susceptibility of those with albinism in Africa is thus well documented, with skin damage developing from a young age. Protection from the sun must start at birth and continue throughout life.

OCA is a stable condition with no cure and requiring lifelong management. Dealing effectively with the health and social issues surrounding albinism in low income countries with limited resources is a challenge. The World Health Organisation recognises albinism as a significant public health issue throughout sub-Saharan Africa and is currently investigating this at-risk population [15]. Studies in Zimbabwe have documented the plight of families affected by albinism [16] but also highlighted the imaginative and effective self-management strategies that have been adopted with little or no access to specialist health care facilities or social welfare support [17].

An earlier study [18] of 38 pupils with albinism attending a well resourced special school educating pupils with visual impairment in a rural area of northern South Africa concluded it provided a 'health promoting' environment. Staff were aware of the special needs of children with albinism and the school timetable and buildings had been adapted to minimise their sun exposure. Furthermore, the government in South Africa provide a generic SPF 15 sunscreen cream free of charge through the regional hospitals. Despite this, all the pupils had sun induced skin damage including sunburn when examined on a sunny day in autumn. Their hats protected only the upper part of the face, leaving the lower face and neck exposed. Clearly the sun protection strategies adopted by these pupils were not effective. This finding motivated the more in depth study at the same school reported here.

In this follow up investigation community based participatory research methods were used to explore with children the kinds and styles of hats they owned, their use of sunscreens and their sun avoidance behaviours. Community based participatory research (CBPR) is a partnership approach that equitably involves those being studied [19]. CBPR enhances understanding of and addresses complex social, structural and physical environmental factors [20] while addressing community concerns, values and cultures [21]. It was therefore an ideal framework to use in a project that focused on children with albinism, allowing the children's voices to be heard and exploring the wider social milieu that is crucial to adequate sun protection.

## Methods

### Setting

The participants attended a special school for visually impaired children in a rural area of Limpopo Province in South Africa, located close to the Tropic of Capricorn at a high altitude (1230 m) with an average 8.5 hours of sunshine a day. Pupils wore their own clothes and were encouraged to wear hats at all times as well as long sleeved protective clothing. The school buildings had covered walkways and trees in the courtyard to provide shaded areas outside. Outdoor activities such as sport and gardening, part of the daily routine, were scheduled at times of day when UVR levels were relatively low. Supportive staff provided a safe haven where children felt accepted, were well cared for and acquired the necessary coping strategies and life skills to enable them to function well in a population group where being black is the norm.

### Participants

The pupils were drawn from a wide, largely rural geographic area of northern South Africa as the school has an excellent reputation in educating young children with albinism. At the time of this study, 85.5% (112 out of 131) of pupils had albinism as this is the chief cause of visual impairment in this region. It was thus the 'norm' to have albinism within this group.

### Permissions

This study was part of a multi-disciplinary study on albinism in the region where ethical approval was obtained through the local educational authorities, as studies were conduced on school premises. Signed consent was obtained from the principal of the school who acted on behalf of parents as the study was conducted on school premises and it is a boarding establishment and the parents were not present. When parents came at the end of term to collect their children, a meeting was held where one of the researchers (PL) outlined the study in the presence of the school nurse who interpreted into the vernacular languages. Consent in this case was verbal.

### Data collection

Community based participatory research methods were used to gather data about the sun protection strategies of children with albinism, and the wider social, structural and health policy environments which might have impacted on these. These included face-to-face interviews with children and with school staff, in addition to field-note observations of the behaviours of children at play. By participating with children and staff on a daily basis, the environmental conditions were qualitatively noted to give context and understanding that interviews alone would not have provided [22]. With the permission of the school authorities 90 children with OCA (40 female and 50 male: age range 9–19, mean age 11.8 years) attending primary education were invited to participate. The remaining 22 pupils were all pre-schoolers and the teachers considered them too young to participate. All participants agreed to provide information about their hats and sunscreens. They were asked to attend a group interview with the hat they were wearing on that day as well as any creams they were currently using for their skin. The face to face interviews were conducted in English in the presence of the resident nurse who interpreted in the vernacular languages if necessary. Small groups of class peers (4–12 participants) were interviewed in the nurse's office, in a friendly atmosphere that encouraged free discussion. As the children boarding at this school were from very rural areas, it was important to know where to target future health promotion strategies. As children with albinism are encouraged to wear a hat at all times when outdoors, they may prefer a range of hats for different occasions (e.g. for football or for church attendance). This would also allow for washing or loss. There are also financial implications for poorer families in providing hats and it was important to ascertain who had provided them. The children were asked how many hats they possessed, who had purchased them and details of where they had acquired the sunscreen preparations. The brim width, style and material of the hat worn by each pupil on the day of the interview were recorded. The SPF factor, active ingredients and other information provided on the sunscreen packaging were documented. The pupils were delighted at the interest shown in them and participated enthusiastically.

## Results

### Hat numbers, styles and wearing behaviours

All but one pupil, an 11 year old female, attended the interview with a hat. This pupil did not posses any hats whereas one nine year old female had a collection of eight hats. When interviewed she wore a fashionable patterned red hat with a wide brim of 7.3 cm. The other pupils all had at least one hat, usually purchased by the mother although in a few cases they were a gift from a grandmother or an aunt. The data in Table 1 show no difference between the sexes in the mean number of hats possessed or in the average width of the brim. The width of the brims ranged from 3.0 to 7.7 cm, with a mean of 5.4 cm. Only four hats (worn by three females and one male) had widths of more than 7.5 cm.

**Table 1:** Hats worn by pupils with OCA

**Group**	**Number of participants**	**Number of hats**	**Mean brim width (cm)**
Female pupils	40	0–8; mean 3.5	5.4
Male pupils	50	1–5; mean 3.1	5.4*
TOTAL	90	Mean 3.3	5.4

* This value excludes the 3 woollen hats with no brim but includes the 9 base ball style caps

Nine males (18% of the male group) wore baseball style hats, which offered good protection to the front of the face with brim widths of 6.5 to 7.3 cm, but none to the neck or side of the face. These males all played soccer in their free time and were severely sunburnt, with the part of the face protected by the cap outlined pale against the surrounding red skin. Two 10 year old boys wore woollen caps with no protective brim. All the other hats were made from cotton or denim. Although the school nurse was well pigmented and not highly susceptible to sun induced damage, she also wore a hat at all times to encourage compliance among the pupils.

### Sunscreen preparations

The children were asked about their sunscreen preparations, and the details of the sunscreen lotions they brought to the interviews were recorded. All the pupils were aware of the need to use 'creams' on their skin but did not know what the term SPF meant. Over a third (37.8%) of participants were using a sunscreen with some level of SPF at the time of interview (Table 2).

**Table 2:** SPF preparations used by pupils with OCA

**Participants**	**Pupils using commercial or ** **SPF preparations provided by** regional hospitals*	**Pupils using government ** **sponsored SPF15 creams**
40 female	15 (37.5%)	6 (15%)
50 male	19 (38.0%)	5 (10%)
90	34 (37.8%)	11 (12.2%)

* A government sponsored generic SPF15 cream was provided free of charge, collected on a 3 monthly basis from regional hospitals

An additional 17 pupils (18.9% of the group) used aqueous creams with no sun protection factor. The remainder did not have any creams. Two 11 year old females said their sunscreens were finished; one said her mother was away and her grandmother who was caring for her could not afford it. The majority (18/24; 75%) of the commercial sunscreens had an SPF value of 30 or more with only three (12.5%) lower than SPF15 (Table 3).

**Table 3:** Product descriptions on commercial SPF preparations used by pupils

**SPF value**	**Number of pupils**		**With UVA + UVB protection**	
	Female	Male	Female	Male

33	1	1	1	0
30 or 30+	6	10	4	6
25	0	1	0	1
15	1	1	0	0
10	1	1	1	1
5	0	1	0	1

**TOTAL**	**9**	**15**	**6**	**9**

In total 24 pupils (26.7% of the group) used commercial sunscreens; 15 of these (62.5%) offered broad spectrum protection against UVA and UVB

An encouraging 62.5% of the sunscreens purchased protected against both UVA and UVB, nine (37.5%) were either 'waterproof' or 'water resistant' and three (12.5%), all with an SFP of 30+, advertised that they were 'ideal for children'. One SPF30 preparation professed to 'block 97% of harmful rays' and a SPF33 Dermasun sunscreen used by a 13 year old female claimed to be 'alcohol free, non toxic and non greasy' as well as giving a website for additional information. A 14 year old male used an SPF25 sunscreen that protected against UVA+B, was 'oil free, non-irritating and moisturising'. Despite this, he was severely sunburnt as a result of playing soccer regularly.

### Uptake of free government SPF15 sunscreen

Tubes of generic SPF 15 cream were available free of charge through local regional hospitals but only six females and five boys (15% and 10% pupils) were using these at the time of interview (Table 2). These tubes contained no product information. Barriers to access mentioned by participants included the time and expense in travelling to these outlets and the limited availability of the creams. Pupils said they were only given a small number of tubes on each visit, usually three, and on occasion there were none at all available.

Although some children in this study came from families who could afford to buy SPF creams, these were unaffordable for those from lower income families. Whereas a quarter (26.7%) used commercial sunscreen preparations only 11 (12.2%) had government sponsored SPF15 cream, a very low uptake.

### Skin monitoring and treatment

Acute effects of sun induced skin damage observed in pupils at this special school were sunburn, blistering, dryness and chapping of the lips, which sometimes led to infection. When interviewed, the school nurse explained that she monitored the pupils' skin on a daily basis during term time and treated any skin lesions. She reported that the pupils suffered constant dryness and chapping to their lips. Split skin on the lips often lead to infections which were treated with antiseptic creams. There were no visits by a dermatologist or other specialist to the school. Consequently, she travelled to the regional hospital with any pupils requiring further treatment which was both time consuming and expensive. The nurse felt it essential that she accompanied the pupils as hospital staff were often ill informed about albinism, feared the condition and refused to touch those affected.

## Discussion

This CBPR follow-up investigation into sun protection strategies found that:

• Pupils possessed sufficient hats, but the brim widths were largely insufficient to protect the lower face and neck;

• Sun exposure was particularly acute during leisure activities, and there is a balance to be found between sun protection and 'normal' childhood activity;

• Whilst children were aware of the need to use sunscreen lotions, they did not understand the term SPF;

• Application of sunscreen lotions was sporadic;

• There was an insufficient supply of sunscreen lotions;

• Cost and access to sunscreen lotions was prohibitive;

• There was little accessible dermatological support.

Although the daily school routine was organised to minimise sun exposure it was more difficult to curtail pupils' exposure during their free recreational time, particularly over the week-ends and during school holidays when pupils returned home, often to rural villages. Previous work has found that hat wearing is not the norm in rural areas and that the leisure activities of boys and girls are different [17]. The boys opted to play soccer out of doors and many suffered repeated sunburn. The girls preferred watching television and games such as skipping, played in the covered corridors or shaded areas of the courtyard. These findings are in line with other work on albinism as a public health issue [15].

An obvious health care strategy would be to deliver a regular supply of sunscreen to the school, to be distributed by the nurse. Regular visits by a dermatologist, rather than each pupil having to travel into the local hospital for treatment of skin lesions, would also be advantageous. The success of the Regional Dermatological Training Centre in Moshi, Tanzania offers an alternative model where a mobile skin care clinic regularly visits rural areas to monitor the skin of people with albinism and offer sun protection advice [23].

Sunscreens do not prevent photodamage, including a variety of molecular changes such as DNA damage and suppression of immune functions [24]. Limiting sun exposure must be the key intervention to prevent these deleterious effects. Furthermore, light scattering in the atmosphere means that vulnerable people can still become sunburnt in the shade. Those with OCA should move indoors at times of peak UV intensity rather than merely seeking the shade. Sun avoidance costs nothing in monetary terms but bears a social cost as it prevents children with albinism from partaking in sport and other social activities. The children with OCA at this special school strived to be 'normal', to play soccer like their dark skinned peers, despite their visual problems which made it difficult for them to follow the ball. The adverse effects of sun exposure have to be balanced against the social isolation if they are barred from participating in these recreational activities.

Wearing a hat is a simple photoprotective measure. Although children at this school were actively encouraged to wear hats, with compliance high, this failed to prevent burning and skin damage. The pupils in this study possessed an average of three hats, with some owning eight, suggesting that families could afford this expense. Cost was not prohibitive as may be the case in other areas of Africa [25]. The fabric of the hats examined in this study was appropriate, as thickly woven cotton and denim transmit little UVR and are the materials of choice for offering sun protection [26]. The mean brim width of 5.4 cm, however, offered limited protection to lower parts of the face and the neck while the baseball caps favoured by the soccer players provided even less. While all hat styles Diffey and Cheeseman [27] examined on model headforms offered good protection to the forehead, those with little (less than 2.5 cm) or no brim provided negligible protection to other parts of the head and neck. They recommended a brim width of at least 7.5 cm to offer reasonable protection to the nose and cheeks.

Although UVB results in the more immediate and obvious results of sun damage by causing sunburn, UVA has long term effects on the skin, causing photoaging [28]. Individuals with OCA should be protected against both. Topical application of a chemical formulation as a physical or chemical screen to absorb, scatter or reflect damaging UVR was used by more than a third (37.8%) of pupils (Table 3), with almost all having an SPF value of 15 or higher and two thirds (62.5%) broad spectrum preparations offering protection against both UVA and UVB. The commercial sunscreens purchased were suitable, suggesting most parents received correct advice from pharmacies as the SPF value remains the gold standard for indicating sunscreen efficacy and the most reliable indicator for the consumer [28]. However, the degree of sunburn in the group suggested they were used erratically and sporadically. Pupils reported often running out of their sunscreen and not applying them every day. They appeared to ration their use, so they would last as long as possible.

The commercial market for sunscreen preparations is wide and bewildering, even after choosing an appropriate SPF product [29]. Resistance to removal by water or sweat is an important aspect of performance [30], especially when playing sports in a hot climate, as there can be significant loss of the product due to sweating. Water resistant SPF 30 preparations would be the products of choice for the soccer players at this special school. Additional product information is useful for selection of an appropriate formulation according to personal preference. For example, ethanol based products can cause dryness and irritation to the skin, leading some consumers to opt for a product that is ethanol free. Others may choose less oily, sticky preparations, making 'oil free' a useful directive on the packaging. The most important factor is to choose a preparation with an SPF of at least 15 that will be used by that individual.

The decision by the government to provide SPF15 cream rather than a higher SPF is not unreasonable, as the increase in effectiveness in blocking UVB increases only marginally from SPF 15 to 30, from 93 to 96% [10]. The key issue here is one of quantity and reliability of supply. The inadequate provision of sufficient SPF15 cream may be due to a lack of understanding of how this is used. The small tubes provided are similar in size to the antiseptic creams used to treat infected skin lesions and are insufficient for regular daily application of protective cream to all sun exposed parts of the body. In this case the right policy decision was made: to provide the cream free, but the actual needs were not explored and hence not addressed. This perhaps highlights a lack of understanding among policy makers and even health care workers with no direct experience or knowledge of the hypopigmented skin. Tubs of cream are required to ensure regular, sustained use, rather than the small tubes on offer as consistent, regular and proper use of sunscreen is more important than a high protection factor. Lip products with a sunscreening agent should also be made available to alleviate the sun induced damage to the lips.

For effective health communication, relevant information must be both accessible and usable. Participants in this study have clearly received the message to reduce their sun exposure and wear a hat at all times, yet are not protected against damaging sun exposure. The sun protection message is insufficiently detailed and may be incorrectly interpreted. Group discussions during this study suggested pupils were wearing hats for two purposes: to protect their skin and to shade their eyes from bright light. Wearing hats pulled low down over their eyes to reduce their photophobia appeared to be the primary motivation for wearing a hat for some pupils. They also seemed to be applying their sunscreens erratically and inappropriately. Further investigation on how they applied their sunscreen and to which sites of their body would be informative. A study in Tanzania concluded some patients had not understood the purpose of applying sunscreen, with 10% also applying it at night [31], highlighting the importance of evaluating health promotional messages.

Many people in rural parts of South Africa do not have access to the internet and at present information on albinism is provided via leaflets in the vernacular languages, obtained from genetic nurses at regional hospitals. It would be useful to assess how accessible and useful these are to different users, primarily females in families affected by albinism, as they are the main carers, but also to health care and educational professionals caring for people with albinism. People with OCA in Tanzania attending an outreach clinic who had read an educational booklet did not have a better understanding of sun avoidance than those who had not [31] suggesting that this format may not be the most effective way of communicating information on the management of albinism.

Rather than a didactic approach where information is delivered in a top-down manner by professionals, we advocate an interactive, social learning approach for outreach programmes, support groups and workshops focussing on the management of albinism. Using people with albinism to deliver information on sun protection, as well as other aspects of albinism, may be more effective and powerful than receiving the message from dark skinned education and health care professionals lacking first hand knowledge of the consequences of living with a hypopigmented skin in a sunny climate. University students in northern South Africa have volunteered to take on this role. Training of this core group to act as peer educators offers a promising way forward.

This was a small study using participatory methods in one rural school in northern South Africa. The findings, whilst consistent with other studies, may not be generalisable to other settings. The participants were all at a boarding school for children with visual impairment and probably received far more supervision and education on sun protection strategies than might be found for children with albinism at other schools. Prevalence studies would be better positioned to provide information about the nature and extent of sun lesions, and randomised controlled trials would be able to test the effectiveness of health promotion interventions. Nonetheless, the descriptions of sun protection strategies afforded by this investigation provide insight into the lives of pupils with albinism, with implications for public health measures across the region.

## Conclusion

Despite a participatory health intervention strategy at this special school where the pupils, staff and parents collaborated in encouraging good sun protection practices, this study revealed that provision of government sponsored SPF cream was not meeting their needs, and sun protection behaviours were not preventing acute skin damage.

The challenge in health education is to empower those with albinism in Africa with the knowledge to manage their condition effectively, to have access to appropriate monitoring and treatment facilities and an adequate supply of sunscreen preparations.

## Competing interests

The authors declare that they have no competing interests.

## Authors' contributions

This paper was prepared and the final version agreed by both authors. PL conducted the investigation in South Africa.

## Pre-publication history

The pre-publication history for this paper can be accessed here:


